# Consumption patterns and eating behaviors of schoolchildren associated with mental health problems: a Brazilian nationwide study

**DOI:** 10.1371/journal.pone.0320127

**Published:** 2025-05-06

**Authors:** Marília Barreto Pessoa Lima Rodrigues, Giovanna Angela Leonel Oliveira, Ariene Silva do Carmo, Jéssica Pedroso da Silva, Eduardo Yoshio Nakano, Vivian S. S. Gonçalves, Natacha Toral

**Affiliations:** 1 Graduate Program in Human Nutrition, University of Brasilia, Brasilia, Distrito Federal, Brazil; 2 Graduate Program in Public Health, University of Brasilia, Brasilia, Distrito Federal, Brazil; 3 Department of Nutrition, University of Brasilia, Brasilia, Distrito Federal, Brazil; 4 Department of Statistics, University of Brasilia, Brasilia, Distrito Federal, Brazil; University of Maribor, SLOVENIA

## Abstract

**Background:**

Children’s health should be analyzed in a broad context that considers different determinants. Few population-based studies have been conducted, especially with children about food consumption and eating behaviors associated with mental health. This study aimed to assess the association of consumption patterns and eating behaviors with mental health problems in Brazilian schoolchildren.

**Method:**

The participants were children between 6 and 11 years old (n = 1967) from Brazilian schools and their parents. The child’s food intake was assessed using the Questionnaire on Food Consumption for Brazilian Schoolchildren (QUACEB), and the Illustrated Questionnaire on Eating and Sedentary Behaviors (QUICAS) was used to assess eating behavior. The child’s mental health was investigated using the Portuguese version of the Strengths and Difficulties Questionnaire. Principal component analysis was performed to identify consumption patterns and eating behaviors.

**Results:**

Children with higher consumption of ultra-processed foods, less healthy food diversity, and unhealthy eating behaviors (eating distractedly with screens, alone, at irregular times, snacks, or processed foods, and not participating in kitchen tasks before or after meals) had a 45% higher chance of having mental health problems (OR 1.45; CI 1.12–1.87).

**Conclusions:**

There is an association between consumption patterns and eating behaviors with mental problems in Brazilian schoolchildren. Our results reinforce the importance of analyzing the set of health determinants.

## Introduction

Changes in health and nutrition profiles are influenced by several factors, including changes in dietary patterns (especially access, availability, type, and marketing of foods), food environment, and public policies. In 2023, data from the Brazilian Food and Nutrition Surveillance System (SISVAN in Portuguese) showed that 29.4% of children between 5 and 9 years of age who used Primary Health Care were overweight, and of these, 17.3% were obese [1]. In parallel, the consumption of processed and ultra-processed foods has increased in Brazil. These represent approximately 50% of the total energy consumed by children [2] and about 20% of the total calories consumed by the Brazilian population 10 years old and above [3,4].

Ultra-processed foods (UPF) have unbalanced nutritional composition, with high sodium, sugar, and fat content; moreover, they are formulated to be extremely tasty, inducing their frequent consumption [5]. They also encourage distracted eating, as they are often consumed standing, without the use of silverware, or even while the person is performing another activity [5]. Thus, consumption of UPF is particularly critical early in life when children are forming their basic eating habits. Childhood is considered a window of opportunity for healthy lifestyle development, as behaviors learned at this stage tend to be perpetuated throughout life [6].

Consumption of UPF is associated with several negative health outcomes. Recent systematic reviews and meta-analyses show that higher consumption of UPF is associated with increased risk of overweight, obesity, metabolic syndrome, dyslipidemia, hypertension, diabetes, cardiovascular disease, and depression in adults, among other diseases, as well as increased risk of all-cause mortality [7–14]. In Brazil, almost 30% of the increased prevalence of obesity between 2002 and 2009 was due to the consumption of UPF [15].

Among children, studies have shown an association between consumption of UPF and higher levels of total cholesterol, LDL cholesterol, triglycerides, and increased waist circumference, which may predisposes individuals to the development of cardiovascular disease [2,16,17].

A systematic review and meta-analysis – including studies with adults, adolescents, and children – also showed an association between consumption of UPF and common mental disorders (depression and anxiety, assessed together or separately) and other mental health problems (such as trauma, stress, food or alcohol dependence, and eating disorders [18]. Worldwide, about 8% of children age 5–9 and 14% of adolescents live with mental health problems [19].

According to the WHO, mental health can be defined as a state of well-being that enables people to cope with life stresses, realize their abilities, learn, and work well, and contribute to their communities. For children, the emphasis is on developmental aspects, such as their ability to manage emotions and thoughts, build social relationships, and learn and acquire knowledge. Therefore, it is more than the absence of a mental disorder. Mental disorder or mental health problems are clinically significant disturbance in an individual’s cognition, emotional regulation, or behavior, usually associated with distress. The most common mental health problems during childhood are conduct problems, attention-deficit/hyperactivity disorder, anxiety, and depression [19].

Mental health problems during childhood or adolescence often tend to persist, and if left untreated, can impair child development, quality of life, negatively affect the family environment, and increase the risk of other problems, including substance abuse and unemployment [20–22]. Evidence shows a continuum of mental health problems from childhood into adulthood; thus, studies that focus on early identification of difficulties related to children’s mental health are important [23].

Studies suggested an association between dietary patterns and mental health problems. In the study by Farhangi, Dehghan and Jahangiry [24] showed that female adolescents in high scores of low fat eating pattern were less likely to have mental disorders. In a cross-sectional study with preschoolers children in China [25] suggest that wider food varieties were associated with better mental health. High consumption of sugar-containing soft drinks and sweets and breakfast skipping were associated with various mental health problems among children and adolescents [26–28].

Studies indicate a significant association between unhealthy eating and worsening mental health in children and adolescents [18,24,29,30]. However, few population-based studies [18,24,29,30], especially with children, have been published that consider a set of risk or protective factors, related to food consumption and eating behaviors, in association with mental health. Thus, the objective was to evaluate the association of consumption patterns and eating behaviors with mental health problems in Brazilian schoolchildren.

## Methods

A cross-sectional study with children, 6–11 years of age, and their parents with access to the Internet. This is part of the “Study of Nutrition of Schoolchildren – ENUCE, approved by the Research Ethics Committee of the Faculty of Health Sciences, University of Brasilia. Only the responses from parents who agreed to the Informed Consent Form and whose children also positively agreed to the Consent Form were included in the study.

### Sample size

The sample was calculated considering the population of 14,533,651 children who study in the first to fifth grade in Brazilian elementary school in Brazil, according to the 2021 National School Census [31]; confidence level of 95%; and 89% prevalence of ultra-processed foods consumption by children 5–9 years old, according to data from SISVAN of 2021 [1]. The absolute error was approximately 0.014; therefore, a minimum of 1,966 children should be assessed. The calculated value of the sample was distributed proportionally to the population according to the five Brazilian macro-regions and the type of the school (private or public), according to the National School Census [31]. The sample obtained should not deviate more than 30% from the distribution in relation to the estimated population (Table 1).

**Table 1:** Sample characterization. Brazil, 2022.

Variables	Population estimated by School Census, 2021 n (%)	Calculated sample n (%)	Divergence
**Brazil**	14,533,651 (100)	1,967 (100)	-30 - + 30
**Macroregions**			
North	1,616,919 (11.12)	285 (14.49)	30
Northeast	4,132,922 (28.44)	486 (24.71)	-13
Central-West	1,167,389 (8.03)	206 (10.47)	30
Southeast	5,660,515 (38.95)	685 (34.82)	-11
South	1,955,906 (13.46)	305 (15.51)	15
**School types**			
Public	11,919,578 (82.01)	1,711 (86.99)	6
Private	2,614,073 (17.99)	256 (13.01)	-28

### Data collection

Data was collected between 02 of February and 17 of November 2022 via an online questionnaire on the Google Forms Platform. Participants were recruited using the snowball technique [32]. The questionnaire was disseminated through the social networks of the researchers involved, the institution, and by e-mail to the state and municipal education departments, nutritionist councils, the union of primary education workers, and private schools in the capital cities throughout Brazil. The parents and children spent around 25 minutes filling out the questionnaire.

### Eligibility criteria

The questionnaire was answered by 2,206 people. The sample excluded 25 responses whose parents had not agreed with the informed consent form; 2 duplicate responses from the same person; 13 children that had not agreed with the informed consent form; 78 children under 6 years and over 11 years old; 81 children who had diseases that could directly or indirectly affect the results of the study, such as diseases of the gastrointestinal tract or that alter food intake, 30 children with previously diagnosed emotional or mental disorders reported by parents, and 10 children with cognitive diseases and conditions.

### Instrument and variables

The questionnaire first contained questions directed to the children’s parents and, later, questions for the child to fill out – without interference – using the same access link. The instructions said that, if necessary, the parent could read the questions to the child but should not influence the child’s answers.

The parent’s questionnaire included three sections: (1) data about the child; (2) mental health of the child; and (3) sociodemographic data. The first section asked the type of the child’s school (public or private), the child’s date of birth, and the following question was added, which was used as an exclusion criterion based on the responses: Does the child participating in the study have any medically diagnosed disease? If so, which disease/condition?. The second section was the Strengths and Difficulties Questionnaire (SDQ). This questionnaire assesses the mental health of children and adolescents between ages 4 and 16. The SDQ was validated for use in Brazil [33]. The questionnaire contains 25 questions answered by parents about the child’s behavior over the past six months, with the response options ranging from “not true”, “somewhat true”, and “certainly true”. In the third section, data about the person responsible were collected, such as age (in years), gender, education level (in categories), and family income (in minimum wage categories).

The child questionnaire included: (1) identification of the child; (2) assessment of food intake; and (3) eating behavior. The first part asked about age (in years), and gender. The next section was the Questionnaire on Food Consumption for Brazilian Schoolchildren (QUACEB in Portuguese). This was previously validated and is an adaptation of a 24-h recall illustrated with 33 food pictures with descriptive captions. The instrument asks the child to mark all the foods consumed the previous day [34]. The last section contains the eating behaviors portion of the Illustrated Questionnaire on Eating and Sedentary Behaviors (QUICAS in Portuguese) [35]. This instrument presents questions, accompanied by pictures, about ten eating behaviors, five of which refer to healthy behaviors (eating without distraction with screens; with company; at regular times; eating real food such as rice, beans, meat, and salad; and participation in meal preparation activities) and five refer to unhealthy behaviors (eating distractedly with screens; eating alone, eating at irregular times, eating snacks or processed foods, and not participation in meal preparation activities). The child was asked to mark which image best described their behavior at main meals (presence or absence of the behavior) the day before completing the questionnaire.

#### Dependent variable.

The dependent variable of the study corresponds to the mental health of the children, as assessed by the SDQ. The questionnaire consists of 25 items, each with three response options: false, somewhat true, and true. “Somewhat true” is scored as 1 point, while “false” and “true” vary depending on the item, scoring either 0 or 2 points. The items related to emotional symptoms, conduct problems, hyperactivity/inattention, and peer problems (20 items in total) are summed to generate a total difficulties score, which ranges from 0 to 40. The items assessing prosocial behavior (5 items) are not included in this score, as they are interpreted separately, reflecting positive aspects of the child’s social and emotional development, unlike the other items which measure difficulties. From the total score, the following classification was adopted: normal (0–13 points), borderline (14–16 points), and child with mental problems (17–40 points) [33]. For analysis purposes, the categories “normal” and “borderline” were grouped together, as adopted by other researchers [36–39].

#### Independent variables.

From the QUACEB data, the following food consumption analyses were performed, considered as independent variables of the study:

(a) NOVA ultra-processed food consumption score – for this analysis, the original simplified ultra-processed food consumption instrument [40,41] was used as a reference. Thus, the QUACEB items corresponding to ultra-processed foods (1– soda; 2– industrialized juices in cartons; 3– chocolate milk; 4– flavored yogurt; 5– packaged bread; 6– packaged salty snacks or crackers; 7– cookie or packaged sweet cake; 8– chocolate, ice cream, gelatin, or candy; 9– salami, sausage, baloney, or ham; 10– margarine; 11– mayonnaise or ketchup; and 12– instant noodles, frozen lasagna, or pizza) were considered for the application of this score. The chocolate milk and flavored yogurt were grouped in a single category called “milk drinks”. The items mayonnaise, ketchup, and margarine were placed in a single subgroup of “sauces”, as suggested by the instrument. Thus, the score was calculated from the sum of subgroups of ultra-processed foods reported as consumed on the previous day: for each “yes” answer, a value of 1 was assigned, and “no” answers a value of 0. Therefore, the score ranged from 0 (none of the foods were consumed the previous day) to 10 (at least one food from each of the 10 subgroups was consumed the previous day).
(b) Diet diversity score – the FAO diet diversity assessment score adapted from Brazilian [40] was used as reference, which assesses only the fresh or minimally processed foods consumed on the previous day. In this score, one point is attributed to the presence of each of the 10 food subgroups: 1– grains and tubers; 2– beans; 3– beef, pork, chicken, fish, or shrimp; 4– eggs; 5– milk; 6– dark green leafy vegetables; 7– fruits and vegetables rich in vitamin A; 8– light green leafy vegetables; 9– other vegetables; and 10– other fruits. Similarly, these are added up for a score that ranges between 0 and 10. Note that rice, cassava/manioc, and potato were grouped in a single subgroup entitled grains and tubers, and the items pumpkin, carrot, mango, and papaya comprised the subgroup of fruits and vegetables rich in vitamin A.

Other independent variables were the child’s eating behavior, assessed by using the prevalence obtained from the QUICAS, and the sociodemographic variables, classified as follows: gender and age of the child; gender and age of the parent, education of the parent (Elementary school or less; High school; Higher education or more), and family income (in minimum wage ranges, considering the minimum wage at the time of the study was R$1,320.00 in Brazilian Reais, equivalent to approximately US$250.00).

### Statistical analysis

Descriptive analysis was done by calculating the distributions of relative frequencies and means with their standard deviation (SD) for categorical and continuous variables, respectively, with their respective 95% confidence intervals. The Shapiro-Wilk Test was used to assess the normality of the continuous variables. The chi-square test and Student’s t-test were used to compare proportions and means, respectively.

Food consumption variables (NOVA ultra-processed food consumption score and food diversity score) and the eating behavior variables (eating distractedly with screens, with company, at regular times, type of food, and participation in meal preparation activities) served as the basis for identifying the patterns used as explanatory variables. The consumption patterns and eating behaviors were obtained from Principal Component Analysis (PCA), which is a multivariate technique. This technique allows the number of variables to be reduced to maximize the explanatory power of the data set. We used the varimax orthogonal rotation method and the Kaiser-Meyer-Olkin (KMO) index to evaluate the factorability of the data, adopting values between 0.5 and 1.0 as acceptable for this index [42] Bartlett’s test of Sphericity was used to test for the adequacy of the correlation matrix. In the standardized estimation analysis, factor loadings greater than |0.3 | and p < 0.05 were considered for the latent variable construct, as an indication that the correlation between the observed variable and the construct was moderately high in magnitude [43]. The number of patterns to be extracted was defined by eigenvalues > 1.0 and the Screen plot criterion. The patterns were generated on continuous variables. The variables in the identified patterns were categorized according to the median reference values of the distribution.

The association of consumption patterns and eating behavior with children’s mental health was initially observed using bivariate logistic regression analysis. Then, multiple logistic regression analysis was performed, considering the adjustment for the sociodemographic variables that presented association (p < 0.05) (Table 3) with the outcome variable of mental health, which were: type of the school, child and parents’ genders, education of the parent, and family income. The Odds Ratio (OR) with 95% confidence interval was used as a measure of association, and p-values <0.05 were considered. Statistical analyses were processed using Stata software version 17.

## Results

The sample consisted of 1,967 dyads of children and parents. The participants were consistent with the distribution of the Brazilian child population across macroregion and school types (Table 1).

The mean score for total difficulties (SDQ) was 10.28 (SD = 6.06), ranging from 0 to the maximum value of 32 points. Of the children, 16.1% (CI 95%: 14.4–17.6) had scores classified as having mental problems, which were more prevalent among males (Table 2).

**Table 2:** Description of sociodemographic characteristics of study participants and association with children’s mental health. Brazil, 2022.

Variables	n	% (CI 95%) or Mean ± SD	Normal or borderline mental health (CI 95%)	Mental problems (CI 95%)	p-value^*^
Sociodemographic data					
**Child’s age**	1967	8.0 ± 1.4	8.0 ± 1.4	8,2 ± 1,5	0.035
**Child’s gender**					
Male	1003	51.1 (48.8-53.2)	81.8 (79.3-84.1)	18.2 (15.8-20.6)	0.007
Female	963	48.9 (46.7-51.1)	86.2 (84.0-88.3)	13.8 (11.6-15.9)	
**Parent’s age**	1877	36.0 ± 7.4	36.2 ± 7.2	34.9 ± 8.3	0.013
**Parent’s gender**					
Male	123	6.3 (5.1-7.3)	91.1 (85.9-96.1)	8.9 (3.8-14.0)	0.027
Female	1841	93.7 (92.6-94.8)	83.5 (81.7-85.1)	16.5 (14.8-18.2)	
**Parent’s education**					
Elementary school or less	528	27.0 (25.0-28.9)	77.7 (74.0-81.2)	22.3 (18.7-25.9)	<0.001
High School	859	44.0 (41.7-46.1)	84.1 (81.5-86.5)	15.9 (13.4-18.4)	
Higher education or more	567	29.0 (27.0-31.0)	89.8 (87.2-92.2)	10.2 (7.7-12.7)	
**Family income** ^**^					
Up to 1 minimum wage	739	38.6 (36.3-40.7)	78.9 (75.9-81.8)	21.1 (18.1-24.0)	<0.001
1 to 4 minimum wages	830	43.3 (41.0-45.5)	85.4 (83.0-87.8)	14.6 (12.1-16.9)	
Above 4 minimum wages	347	18.1 (16.3-19.8)	92.2 (89.3-95.0)	7.8 (4.9-10.6)	

*Note: Data presented as relative frequency (%) and mean ± standard deviation (SD) for categorical and quantitative variables, respectively.

*Simple Student’s T-test and Chi-square test for comparison of means and proportions, respectively.

**Considering the minimum wage at the time of R$1,320.00 reais, equivalent to approximately US$ 250.

An association was identified between children’s mental health and the sociodemographic variables evaluated in the study (p < 0.05). Children who had mental problems had a higher mean age compared to children classified as borderline or normal mental health (p < 0.05). The parents of the children who had mental problems had lower mean age, lower education, and lower income (p < 0.05). In addition, a higher prevalence of mental problems was observed among children whose responding parent was female (p < 0.05).

Children with mental problems consumed more ultra-processed foods and less food diversity (Table 2). All the eating behaviors evaluated, except for eating with company, were associated (p < 0.05) with mental problems (Table 3).

**Table 3:** Description of study participants’ food intake and eating behaviors and association with children’s mental health. Brazil, 2022.

Variables	n	% (CI 95%) or Mean ± SD	Normal or borderline mental health	Mental problems	p-value^*^
**Food intake** NOVA score of Ultra-processed food consumption^1^	1967	3.4 ± 2.0	3.4 ± 2.0	3.7 ± 2.1	0.023
Food Diversity Score^1^	1967	5.1 ± 2.0	5.1 ± 1.9	4.8 ± 2.1	0.010
**Eating behavior**					
**Distractedly with screens at the main meals**					0.001
Ate without watching TV and without touching a tablet or cell phone	1034	52.57 (50.3-54.7)	86.6 (84.4-88.6)	13.4 (11.3-15.5)	
Ate while watching television and playing on a tablet or cell phone	933	47.43 (45.2-49.6)	81.0 (78.5-83.5)	19.0 (16.4-24.4)	
**Company at the main meals**					0.951
Ate with at least one person	1844	93.75 (92.6-94.8)	83.9 (82.2-85.6)	16.1 (14.3-17.7)	
Ate alone	123	6.25 (5.1-7.3)	83.7 (77.1-90.3)	16.3 (9.6-22.8)	
**Regularity at the main meals**					<0.001
Ate at a regular time	1681	85.46 (83.9-87.0)	85.7 (83.9-87.3)	14.3 (12.6-16.0)	
Ate at irregular times	286	14.54 (12.9-16.0)	73.8 (68.6-78.9)	26.2 (21.0-31.3)	
**Type of meals at the main meals**					0.001
Ate real food (like rice, beans, meat, and salad)	1863	94.71 (93.7-95.7)	84.6 (82.9-86.2)	15.4 (13.7-17.0)	
Ate snacks or processed foods	104	5.29 (4.2-6.2)	72.1 (63.3-80.8)	27.9 (19.1-36.6)	
**Participation in meal preparation activities**					0.009
Participated in kitchen tasks	667	33.91 (31.8-36.0)	87.0 (84.3-89.5)	13.0 (10.4-15.6)	
Did not participate in kitchen tasks	1300	66.09 (63.9-68.1)	82.4 (80.3-84.4)	17.6 (15.5-19.6)	

Note: Data presented as relative frequency (%) and mean ± standard deviation (SD) for categorical and quantitative variables, respectively.

*Simple Student’s T-test and Chi-square test for comparison of means and proportions, respectively.

^1^Score range from 0 to 10

The analysis of the PCA allowed the identification of only one principal component, which contributed 22.7% of the variance of the total information (Table 4). The KMO index (0.580) and the factor loadings of all the consumption and eating behavior variables (factor loading > |0.3|) were satisfactory (Table 3). Thus, the identified pattern was characterized by a higher NOVA score of consumption of ultra-processed foods (factor loading = 0.323), lower food diversity score (factor loading = -0.541), and presence of unhealthy eating behaviors, which were eating distractedly with screens (factor load = 0.569), alone (factor load = 0.339), at irregular times (factor load = 0.556), eating snacks or processed foods at main meals (factor load = 0.534), and not participating in in kitchen tasks before or after meals (factor load = 0.400) (Table 4). The Bartlett’s test of Sphericity is highly significant at p < 0.001 which shows that the correlation matrix has significant correlations among at least some of the variables.

**Table 4:** Factorial loadings of observed variables of children’s food intake and eating behaviors make up the construct of principal component analysis. Brazil, 2022.

Observed variables of food intake and eating behaviors	
**Food intake**	
NOVA score of Ultra-processed food consumption	0.323
Food Diversity Score	-0.541
**Eating behaviors**	
Distractedly with screens	0.569
Company	0.339
Regularity	0.556
Type of meals	0.534
Not participation in meal preparation activities	0.400
**Eigenvalues**	1.59
**Explained variance**	22.7%
**Kaiser-Meyes-Olkin (KMO)**	0.580
**p-value for Bartlett’s Test of Sphericity**	<0.001

*Factor load > |0.3 | were considered for the logistic regression analysis.

In simple logistic regression, children with an unhealthy eating pattern and eating behavior had 58% higher odds of having mental problems (OR 1.58; CI1.23–2.01). In the adjusted model, children who had higher scores for unhealthy eating patterns and eating behavior characterized by higher NOVA score for consumption of ultra-processed foods, lower food diversity score, and presence of unhealthy eating behaviors were 45% more likely to have mental problems (OR 1.45; CI 1.12–1.87) (Table 5).

**Table 5:** Crude and adjusted logistic regression model for mental health in children. Brazil, 2022.

Variables	OR crude (95%CI)	p-value	OR adjusted (95%CI)^*^	p-value
Unhealthy consumption patterns and eating behavior ^a^				
<median	Ref.	-0.541	Ref.	-0.541
≥ median	1.58 (1.23-2.01)	<0.001	1.45 (1.12-1.87)	0.004

OR: Odds Ratio; CI: Confidence Interval; Ref: reference category.

^a^Consumption pattern and eating behavior characterized by a higher NOVA score for consumption of ultra-processed foods, a lower score for the diversity of foods, presence of unhealthy eating behaviors, such as eating distractedly with screens, alone, and at irregular times, eating snacks or processed foods at main meals and not participating in in kitchen tasks before or after meals.

*OR adjusted for administrative type of the school, child’s gender, gender of the child’s parent, education of the child’s parent, and family income.

p < 0.001

## Discussion

This study found the association between consumption patterns and eating behaviors of Brazilian schoolchildren and mental problems. Findings revealed that children consuming higher amounts of ultra-processed foods and exhibiting unhealthy eating behaviors—such as eating distractedly with screens, eating alone, at irregular times, consuming snacks or processed foods during main meals, and not participating in meal preparation—were 45% more likely to experience mental health problems.

In our sample, the prevalence of mental problems in schoolchildren was 16.1%, which is a similar prevalence to that found by Maison *et al.* (2020) [44]. In their data from a cohort in the Pelotas, RS, which is located in southern Brazil, the authors observed that the prevalence of mental problems was 10.4% at age 6 and 14.2% at age 11, which translates into a 50% increase between the two time points [44]. Our study found that the mental problems is more prevalent among males, as pointed out by Bach *et al.* [37], who found a 30.0% prevalence among boys and 28.2% among 7- and 8-year-old girls in the southeastern region of the country [37]. Both studies used the SDQ total score to assess mental health. The inclusion of children of different ages, the school characteristics, and the sociodemographic characteristics of the samples might explain the different prevalences found in the studies. Despite such differences, our findings indicate an early onset of mental problems in schoolchildren in elementary school, especially among boys.

Our study showed an association between child with mental problems and the sociodemographic variables evaluated. The children who presented mental problems had a higher average age. The parents who completed the survey of children with mental problems were more often female, had a lower average age, low education, and low income. Similarly, a study conducted in Recife, PE, Brazil, indicated that children with more mental health problems often had mothers under 30 years of age, with less education (9 or fewer years of study), and with family income lower than one minimum wage per month [36]. Another study showed that Chinese children and adolescents with caregivers with low education and low family income were more prone to mental health problems [39]. Bach *et al.* [37] stated that belonging to economically disadvantaged strata increased by 71% the likelihood of emotional and behavioral problems (assessed using the SDQ questionnaire) among schoolchildren [37]. In addition, a meta-analysis study found low socioeconomic status to be an important risk factor for mental health problems (specifically conduct problems) in Brazilian children [45].

Our study showed that children with unhealthy food consumption – a higher NOVA score for consumption of ultra-processed foods and a lower food diversity score – were more likely to have mental problems. A systematic review (with studies from Australia, the United States, the United Kingdom, Germany, China, Canada, and Norway) including 82,779 participants ranging in age from 4.5 to 18 years demonstrated consistent associations between unhealthy eating patterns and worse mental health during childhood or adolescence [25]. Similarly, meta-analysis data on 891,723 adults with a mean age of 39 years found that higher consumption of ultra-processed foods significantly increased the risk of depression [14]. A systematic review and meta-analysis with 385,541 participants – including children (with a mean age of 9.6 years) – evidenced a bidirectional association between consumption of ultra-processed foods and mental health (including depression, anxiety, trauma and stress, addiction, and eating disorders) [19]. Regarding the consumption of fast food, which is mostly unhealthy foods, an association was found between the consumption of these foods and increased chances of mental health problems among children and adolescents 6–18 years of age in Iran [46].

The Dietary Guidelines for the Brazilian population use the NOVA classification of foods as a reference, with emphasis on the group of ultra-processed foods discussed in this article. Although the Guidelines is not restricted to the nutrients in the food, part of the association between the consumption of ultra-processed foods and mental health can be explained by the nutrient profile of these foods and the presence of non-nutritive components used or produced in the manufacturing process of ultra-processed foods. Preclinical and clinical studies suggest a possible role of the emulsifier carboxymethylcellulose, used as an antimicrobial agent, in the link between ultra-processed foods and mental health problems [47]. Another study also suggests that a higher intake of artificial sweeteners (aspartame, saccharin) and monosodium glutamate – substances commonly present in ultra-processed foods – may be related to the dysregulation of the synthesis and release of neurotransmitters implicated in mood disorders, such as dopamine, norepinephrine, and serotonin[48].

In our studies, children in the normal or borderline mental health group presented higher average food diversity scores than those in the mental problems group. However, few studies have examined the association between healthy food consumption and children’s mental health. A meta-analysis of eighteen studies with the adult population demonstrated that high consumption of fruits and vegetables was significantly associated with a reduced risk of depression [49]. For the adolescent population, a systematic review found a positive association between fruit and vegetable consumption and mental health [50]. Renzaho *et al.* [51] in a cross-sectional study of Australian children and adolescents 4–12 years of age showed that fruit and vegetable consumption was associated with protection against mental health problems (assessed by the SDQ questionnaire, specifically on emotional problems) [51].

In our study, children who exhibited an unhealthy eating behavior pattern (eating distractedly with screens, alone, at irregular times, and snacks or processed foods and not participating in kitchen tasks before or after meals) were more likely to have mental problems. Positive associations have been found between screen time and mental problems among children and adolescents, such as in the study of Chinese preschool children 3–6 years of age, which indicated that high screen time (of at least 2 hours per day) significantly increased the risk of mental health problems [52]. Song *et al.* [39] showed that children and adolescents, with an mean age of 11 years, with extensive screen exposure (greater than 2 hours per day) were more likely to have mental problems (assessed by the SDQ) than those with low screen exposure (time less than or equal to 2 hours per day [38]. Furthermore, Booker *et al.* [53] indicated that greater screen use was associated with higher odds of mental health problems among adolescents aged 10–15 years in a longitudinal study in the UK. Our findings also point to extensive screen time adversely affecting children’s mental health.

Similar to our findings, a study of Iranian adolescents 15–17 years old found a significant relationship between unhealthy eating behaviors, such as skipping meals, consumption of snacks like cookies, and frequent eating in fast-food type restaurants, and a higher prevalence of mental health problems [29].

Other studies suggest that regular meals in company of family are a protective factor for mental health in children and adolescents [54,55]. A prospective study conducted in 2016 with public school students in the city of Duque de Caxias, RJ, Brazil, showed that the absence of family meals was associated with a higher frequency of common mental disorders both at baseline and follow-up in a cohort of 2,511 schoolchildren (aged 9–11 years) and adolescents (aged 12–17 years) [56].

Our study presents a joint approach to the components of consumption patterns and eating behaviors from a sample of Brazilian children. Thus, the pattern characterized as unhealthy – higher consumption of ultra-processed foods, lower consumption of food diversity, unhealthy eating behaviors (eating distractedly with screens, eating alone, eating at irregular times, consuming snacks or processed foods, and not participating in kitchen tasks before or after meals) – was associated with higher odds of mental problems in children. Our results reinforce the importance of analyzing the set of health determinants.

Previous studies that have analyzed the contention between consumption patterns, eating behaviors, and mental health are limited and generally evaluate these behaviors in isolation, as discussed earlier. In addition, there is a lack of such studies about the age range of our study (elementary schoolchildren). A recent study evaluated the association between lifestyle patterns and the occurrence of common mental disorders in a robust sample of Brazilian adolescents [57]. The predominantly healthy pattern, characterized by lower consumption of ultra-processed foods, higher consumption of fresh and minimally processed foods, higher water intake, and sufficient physical activity, presented a lower chance of common mental disorders. Another study showed that a healthy eating pattern, characterized by the consumption of vegetables, beans, cereals, and meats, is associated with a lower chance of common mental disorders in adolescents [58]. Furthermore, eating behaviors, such as regular breakfast consumption and regular eating with the family, are associated with a lower chance of common mental eating disorders [58].

High intake of ultra-processed foods and low dietary diversity were associated with a higher chance of mental problems, indicating that interventions aimed at nutritional education and the promotion of healthy eating habits may be strategies for preventing mental health problems in children. In addition, promoting healthy eating behaviors, such as involving children in meal preparation and reducing screen time during meals, may act as a protective factor. The findings point to the need for an integrated, holistic and preventive approach in the formulation of public health policies that consider not only nutrition, but also the social and behavioral context of children.

Important strengths of our study include the selection of a national sample with the inclusion of food intake, eating behaviors, and mental health data of elementary schoolchildren in the same study. Research exploring the aspects studied here, specifically in this age group, is scarce not only in Brazil but also in other countries, which makes this data innovative. The robust statistical analysis with PCA is another noteworthy strength of the present study. In addition, the study addressed the topic of mental health among children during the Covid-19 pandemic, a period that possibly had a significant impact on the mental health of various age groups, due to changes in life routine, including changes in diet and daily school and domestic activities.

However, some limitations also need to be considered. First, due to pandemic conditions, the survey was conducted online, which may have made it difficult for parents and children to understand the questionnaire. Thus, it was impossible to guarantee that they fully understood the applied questions. However, several national and international studies were conducted during this period using the same method [59–61]. The food consumption questionnaire evaluated is a marker of food consumption relative to the previous day, which does not necessarily represent the analysis of usual consumption. However, several studies have used similar questionnaires in comprehensive surveys [41,61], and a validated instrument for consumption assessment has been adopted [17,61]. Due to the cross-sectional design of the study, it is not possible to establish causal relationships between the variables studied.

## Conclusion

The results presented in this study demonstrate an association between consumption patterns and eating behavior characterized as unhealthy, with increased chances of mental problems in schoolchildren. Children with higher consumption of ultra-processed foods, less healthy food diversity, who eat distractedly with screens, alone, at irregular times, opting for snacks or processed foods, and who do not participate in kitchen tasks had a 45% higher chance of having mental problems. Thus, in addition to investigating isolated characteristics, it is important to evaluate the interaction between them. Thus, public health policies should take a comprehensive look at health risk factors to enable more successful interventions regarding children’s mental health. In Brazil, the National School Feeding Program (PNAE) could be expanded to further limit ultra-processed foods in school meals and emphasize fresh and minimally processed foods. Additionally, public health campaigns could focus on educating families about the importance of shared meals, limiting screen time during meals, and involving children in food preparation, as these behaviors were shown to be associated with better mental health outcomes. Other examples aligning with the study`s findings applied public policies in other countries are include taxation on sugar-sweetened beverages in Mexico, which has led to a reduction in the consumption of sugary drinks and could be adapted for ultra-processed products. Additionally, in Finland, mental health programs are integrated into the school curriculum, provide preventive mental health support to children from an early age. Thus, the results indicate the importance of public policies to promote adequate and healthy food for children, considering the association between food and mental health.

## Supporting information

S1 Data Database 2. (XLSX)
